# Hyperactivity in Mice Induced by Opioid Agonists with Partial Intrinsic Efficacy and Biased Agonism Administered Alone and in Combination with Morphine

**DOI:** 10.3390/biom13060935

**Published:** 2023-06-02

**Authors:** Agnes Acevedo-Canabal, Travis W. Grim, Cullen L. Schmid, Nina McFague, Edward L. Stahl, Nicole M. Kennedy, Thomas D. Bannister, Laura M. Bohn

**Affiliations:** Department of Molecular Medicine, The Herbert Wertheim UF Scripps Institute for Biomedical Innovation and Technology, Jupiter, FL 33458, USA

**Keywords:** GPCR, biased agonism, oliceridine, partial agonist, striatum, SR-17018, opioid

## Abstract

Opioid analgesics such as morphine and fentanyl induce mu-opioid receptor (MOR)-mediated hyperactivity in mice. Herein, we show that morphine, fentanyl, SR-17018, and oliceridine have submaximal intrinsic efficacy in the mouse striatum using ^35^S-GTPγS binding assays. While all of the agonists act as partial agonists for stimulating G protein coupling in striatum, morphine, fentanyl, and oliceridine are fully efficacious in stimulating locomotor activity; meanwhile, the noncompetitive biased agonists SR-17018 and SR-15099 produce submaximal hyperactivity. Moreover, the combination of SR-17018 and morphine attenuates hyperactivity while antinociceptive efficacy is increased. The combination of oliceridine with morphine increases hyperactivity, which is maintained over time. These findings provide evidence that noncompetitive agonists at MOR can be used to suppress morphine-induced hyperactivity while enhancing antinociceptive efficacy; moreover, they demonstrate that intrinsic efficacy measured at the receptor level is not directly proportional to drug efficacy in the locomotor activity assay.

## 1. Introduction

Opioid analgesics such as morphine induce hyperlocomotion in addition to antinociception in rodents [1,2,3]; both effects result from MOR activation, as demonstrated by the lack of response to opioid agonists found in MOR-KO mice [4,5]. The MOR is a G protein-coupled receptor (GPCR) primarily known for activating inhibitory Gα_i/o_ proteins. It also interacts with scaffolding proteins, such as βarrestins, to regulate and modulate receptor activity. Previous work using βarrestin2-knockout (βarrestin2-KO) mice showed that morphine induced greater antinociception in the absence of βarrestin2, with less development of tolerance [6,7]. The βarrestin2-KO mice also displayed less morphine-induced hyperactivity [8,9]. Recently, we described a series of structurally related MOR-selective agonists that displayed a preference for inducing MOR-G protein signaling over βarrestin2 recruitment [10]. From this series of compounds, SR-17018 was shown to produce antinociception with minimal respiratory suppression in mice [10,11] and Rhesus monkeys [12]. Moreover, chronic treatment with SR-17018 did not produce tolerance in the hot plate, formalin, or paclitaxel-induced neuropathic pain models in mice [13,14].

In this study, we sought to compare the effects of the noncompetitive biased MOR agonists to clinically used opioid agonists with different degrees of intrinsic efficacy. SR-15099 and SR-17018 were selected because they showed a wide degree of “bias” between G protein signaling (^35^S-GTPγS binding) and βarrestin2 recruitment (enzyme fragment complementation, EFC), and because they were shown to be readily delivered to the brain following systemic injection and have a half-life of over 6 h in mice [10]. Recently, we showed that these compounds are noncompetitive agonists at MOR using ^3^H-diprenorphine and ^3^H-naloxone radioligand binding assays and mouse brainstem GTPγS binding assays [15]. In that same study, we showed that oliceridine, which has been reported to display a preference for G protein signaling (cAMP accumulation studies) over βarrestin2 (EFC) recruitment [16], is competitive with DAMGO [15]. The present study was undertaken to determine whether G protein signaling-biased agonists produce hyperactivity and if co-administration with morphine would produce additive or competitive effects.

Herein we show that the efficacy observed in G protein signaling (^35^S-GTPγS binding in striatum) does not predict the propensity to induce maximal hyperactivity. While they are as efficacious as morphine and fentanyl in striatal membrane GTPγS binding assays, SR-15099 and SR-17018 produce very little hyperactivity, in contrast to morphine and fentanyl. Moreover, despite having the lowest intrinsic efficacy of the group, oliceridine produces peak hyperactivity to a similar degree as fentanyl and morphine. In combination with morphine, low doses of oliceridine lead to increases in hyperactivity. However, SR-17018 attenuates morphine-induced hyperactivity while potentiating antinociceptive efficacy. Overall, we describe a paradoxical relationship wherein the noncompetitive G protein biased agonist, SR-17018, can differentially modulate morphine-induced behaviors in vivo.

## 2. Materials and Methods

### 2.1. Animal Care and Use

Adult C57BL/6J and MOR-KO mice were purchased from The Jackson Laboratory (C57BL/6J Stock No: 000664 and MOR-KO ((B6.129S2-Oprm1*^tm1Kff^*/J) Stock No: 007559) and propagated by homozygous breeding. A total of 613 male C57BL/6J, 37 female C57BL/6J, and 44 male MOR-KO mice were utilized for the studies. All experiments used naïve adult mice aged 10–16 weeks. For male mice, weights ranged between 23 and 35 g, and for females, it ranged from 17 to 26 g. Male and female C57BL/6J experiments were separated and presented accordingly where indicated. Approximately 80% of the C57BL/6 mice were acquired from Jackson Labs, while the remaining 20% originated from the lab’s vivarium space. The mice were group-housed (3–5 mice per cage) with 1/4″ corncob bedding and maintained on a 12 h light/dark cycle with food and water ad libitum. The numbers of mice used per treatment and experiment are included in the figure legends. The use of all mice followed the National Institutes of Health Guidelines for the Care and Use of Laboratory Animals, with approval by UF Scripps Biomedical Research Animal Care and Use Committee.

### 2.2. Compounds

Morphine sulfate pentahydrate was acquired from the NIDA Drug Supply Program or purchased from Millipore Sigma or Spectrum Chemicals. Fentanyl citrate, naloxone hydrochloride dihydrate, and dextroamphetamine (*d*-AMPH) hemisulfate were purchased from Millipore Sigma. Oliceridine hydrochloride (TRV-130) was purchased from MedKoo Biosciences. SR-17018 and SR-15099, as mesylate salts, were synthesized at Scripps Research as previously described and validated by NMR for purity greater than 95%. DAMGO [D-Ala2, N-Me-Phe4, Gly5-ol]-Enkephalin (trifluoroacetate salt) was purchased from Tocris. For the in vitro studies, test compounds were prepared as 10 mM stocks in 100% dimethyl sulfoxide (DMSO, Thermo Fisher Scientific, Waltham, MA, USA) and stored at −20 °C in 10 µL aliquots to avoid repeated freeze–thaw cycles, while DAMGO was prepared as 10 mM stocks in sterile water. SR-17018 is difficult to solubilize; therefore, extra care was taken with DMSO to assure purity. Since DMSO is hygroscopic, it was aliquoted in glass bottles and kept at 4 °C before use (pure DMSO is solid at 4 °C).

### 2.3. Drug Solutions Preparation

All experiments were conducted by investigators blinded to treatment assignments by a different experimenter. SR compounds were dissolved from a powder immediately before use in vehicle 1:1:8 (10%DMSO (first):10%Tween-80 (second): then 80% sterile water) and administered intraperitoneally (i.p.). Conventional opioids and *d*-AMPH were either made in the 1:1:8 vehicle or saline, as indicated. Morphine sulfate, fentanyl citrate, oliceridine, naloxone, and *d*-AMPH solutions were prepared based on the salt weight of the drug, while the mesylate salts used for the dosing of the SR compounds were adjusted for the free base weight as previously described [10,13]. All compounds were prepared right before use and administered at volumes of 10 μL/g mouse weight. For co-administration studies, two consecutive injections were given on alternating sides.

### 2.4. ^35^S-GTPγS Binding Assay in Mouse Striata

The striatal membrane G protein coupling assays were conducted as previously described [10,15,17], with minor modifications. Striata isolated from untreated C57BL/6J (10–16 weeks old) male mice were homogenized by a tissue tearer and glass-on-glass homogenization in membrane buffer (10 mM Tris-HCl pH 7.4, 100 mM NaCl, 1 mM EDTA), passed through a 26-gauge needle (insulin syringe) 8 times, and resuspended in assay buffer (50 mM Tris-HCl pH 7.4, 100 mM NaCl, 5 mM MgCl_2_,1 mM EDTA, and 20 μM GDP). For each reaction, 2.5 μg of membrane protein per well was incubated in assay buffer containing ~0.1 nM of ^35^S-GTPγS, with increasing concentrations of the agonist to a total volume of 200 μL, for 2 h at room temperature. The reactions were terminated through filtration with GF/B filters (PerkinElmer, Waltham, MA, USA) using a 96-well plate harvester (Brandel Inc., Gaithersburg, MD, USA). Filters were dried overnight, and radioactivity counts were measured using a TopCount NXT HTS microplate scintillation and luminescence counter (PerkinElmer). In all cases, assays were performed in duplicate or triplicate, and technical replicates were averaged into single data points before combining assays between days to generate means with errors. The EC_50_, IC_50_, and E_max_ values were determined via nonlinear regression using GraphPad Prism 9.0 software when convergence was obtained. Each n represents 1 mouse.

### 2.5. Locomotor Activity

The Versamax Animal Activity Monitoring System [20 × 20 cm^2^] (Accuscan Instruments, Columbus, OH, USA) was used to assess open field locomotor activity as previously described [8]. The system consisted of a photocell-equipped automated open field chamber contained inside sound-mitigating boxes to record locomotor activity. The Versamax open field activity monitor (20 × 20 cm^2^, Accuscan Instruments, Columbus, OH, USA analyzed with Versadat Software v. 2.61) recorded the total number of beam breaks made per animal, and these were collected in 5 min intervals. Mice were individually placed into the activity monitoring boxes for 30 min to habituate (except where indicated) to the new environment, and their basal activity was recorded. Following habituation, for each drug tested, the recording of activity was paused and animals were removed, injected, and immediately put back into the activity boxes to continue to monitor opioid-induced locomotor activity over the indicated times.

### 2.6. Opioid–Opioid Interactions in the Acute Thermal Antinociceptive Responses

Antinociceptive responses to thermal stimuli were determined according to previously published protocols [10]. Mice were placed in a Plexiglass chamber (16″ tall, 8″ in diameter) on a ceramic plate heated to 52 °C (hot plate test; Hot plate Analgesia Meter, Columbus Instruments, Columbus, OH, USA). Basal nociceptive responses were determined by timing the amount of time until a mouse licks or flutters its fore- or hind-paws, rapidly steps, or jumps. To avoid tissue damage, we imposed a ceiling time of 20 seconds for the hot plate. Antinociceptive responses were collected at the indicated time points immediately following injection. Data are presented as “% maximum possible effect”, which was calculated by (response latency − baseline)/(maximal response cutoff latency − baseline) × 100%. 

### 2.7. Software and Statistical Analysis

Data are presented as the average of the mean ± SEM or as the mean with 95% confidence intervals, as indicated. Data were analyzed utilizing GraphPad Prism version 9.1 (for Mac, GraphPad Software, San Diego, CA USA). For GTPγS binding, the radioactivity counts were normalized to the baseline, and a maximum response produced by DAMGO and a 3-parameter nonlinear regression analysis was applied to obtain the efficacy and potency of the compounds. Two-way repeated measures ANOVA tests were used to compare time course-dependent dose effects, and an ordinary one-way ANOVA test followed by a Šídák’s multiple comparison post hoc analysis were used to compare the total distance traveled for drug effect (sums). The two-way RM-ANOVA results are shown as data table inserts into figures, and the results of the one-way ANOVA are indicated by symbols in the figures. Significance was determined using alpha = 0.05.

## 3. Results

### 3.1. Evaluation of the Intrinsic Efficacy and Competitive Nature of Partial Agonists in Mouse Striatal Membranes

**Figure 1:**
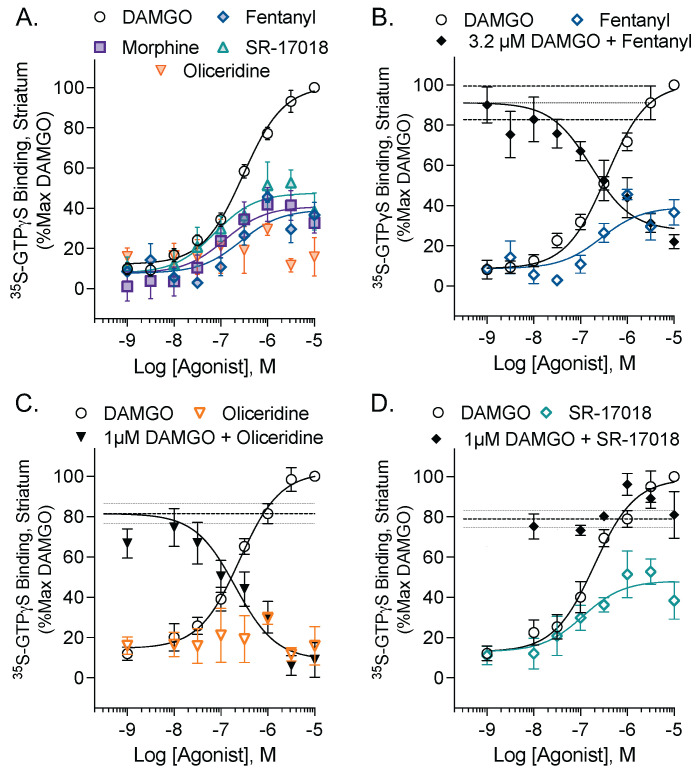
Evaluation of intrinsic efficacy of partial agonists of MOR in mouse striatum membranes by ^35^S-GTPγS binding. (**A**) Morphine (Emax 41 (35–48)%; EC_50_:122 (50–298) nM, *n* = 9), fentanyl (Emax: 39 (31–47)%; EC_50_:151 (56–358) nM, *n* = 6); oliceridine (EC_50_: not converged, *n* = 4), and SR-17018 (Emax: 48 (48–58)%, EC_50_: 119 (105–128) nM, *n* = 5), are partial agonists relative to DAMGO (Emax: 100%, EC_50_ 290 (218–394) nM, *n* = 13). Partial agonist competition with full agonist activity can be observed for (**B**) fentanyl (+3.2 µM DAMGO: IC_50_: 171 (50–520) nM, *n* = 3) and (**C**) oliceridine (+1 μM DAMGO: IC_50_ 203 (95% CI: 79–500) nM, *n* = 4), but not for (**D**) SR-17018. DAMGO was run in parallel on each plate, and IC_50_ calculations were constrained to the % stimulation obtained by DAMGO at the competitive dose on the plate; the mean of all the DAMGO curves is shown in A. The mean DAMGO stimulation was 1.41 ± 0.03 times higher than the baseline (1206 ± 252 cpm). Data are presented as mean ± SEM and 95% CI in the graphs and parameters.

DAMGO, an enkephalin analog, is a full agonist for stimulating ^35^S-GTPγS binding in mouse striatal membrane preparations, while morphine, SR-17018, and fentanyl produce submaximal stimulation (Figure 1A). Oliceridine and fentanyl are known agonists at MOR; however, in this endogenous setting, they produced no measurable stimulation (oliceridine) or only modest stimulation (fentanyl). A low-efficacy agonist will antagonize the effects of a full agonist down to the level of activation that the partial agonist produces; stimulation with 3.2 µM DAMGO provided a large enough window for inhibition by fentanyl (Figure 1B). Since oliceridine did not produce a measurable stimulation on its own, a lower (1 µM) concentration of DAMGO could be used for the inhibition curve. This was to ensure that the oliceridine inhibition curve was not excessively shifted rightward (Figure 1C). Although SR-17018 is also a partial agonist (48%) in striatum, it is noncompetitive, as it did not antagonize a 1 µM stimulation by DAMGO in the striatal membranes (Figure 1D). This agreed with the observations made in MOR-expressing CHO cells and in the mouse brainstem, where SR-17018 was characterized as a noncompetitive agonist by competition studies using functional assays and radioligand binding; this work also showed a lack of agonist-induced GTPγS binding in MOR-KO mouse brainstem membranes [15]. Taken together with the prior study, SR-17018 acts as a noncompetitive partial agonist at MOR, whereas oliceridine and fentanyl act as competitive partial agonists in the mouse striatum and brainstem.

### 3.2. Comparison of Locomotor Activity by Different Opioid Agonists

**Figure 2:**
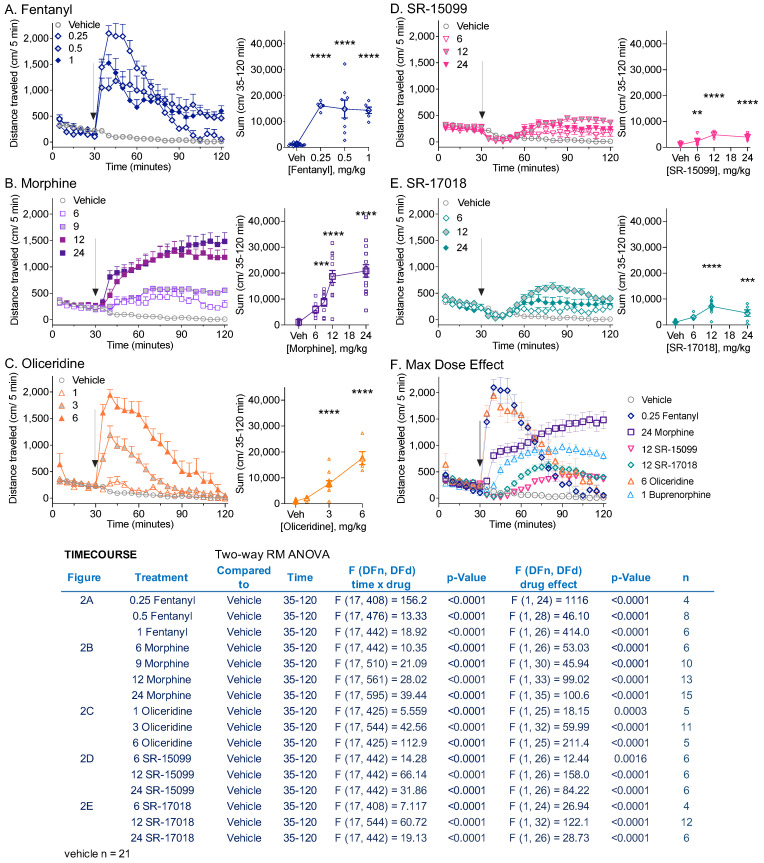
Comparison of locomotor activity by different opioid agonists. Locomotor activity using the open field locomotor boxes, measuring total distance traveled (cm/5 min) with increasing doses of (**A**) fentanyl, (**B**) morphine, (**C**) oliceridine, (**D**) SR-15099, and (**E**) SR-17018 in C57BL6/J males. A vehicle cohort is shown for comparison between each group. Basal locomotor activity was recorded for 30 min, followed by injection with the vehicle or test compound, and activity was monitored for 90 additional minutes. All compounds were dissolved in vehicle 1:1:8 (10%DMSO:10%TWEEN80:80% sterile water). (**A**–**E**) Time course of total distance traveled (cm/5-min) (**left**) and total distance sums after treatment (cm/35–120 min) (**right**) are presented. Doses are indicated in the figure (mg/kg, i.p.). Two-way RM-ANVOA analyses for the time course data are presented in the statistical table within the figure. The figures denoting the sum of distance traveled are shown as individual sums per mouse and as mean ± SEM. For the sums, statistical comparisons to the vehicle was performed by ordinary one-way ANOVA, followed by Dunnet’s post hoc comparison test (** *p* < 0.01; *** *p* < 0.001; **** *p* < 0.0001). (**F**) Time course comparison of the maximum dose effect of all tested compounds. Data are presented as mean ± SEM of the total distance (cm/5 min).

Fentanyl, morphine, and oliceridine induced hyperactivity relative to the vehicle (Figure 2A–C, interaction of time and treatment, *p* < 0.0001) as previously reported [1,2,3,18]. SR-17018, as well as the structurally related partial and biased noncompetitive agonist, SR-15099, which was shown to be a noncompetitive partial agonist in transfected cells and in the mouse brainstem [15], also produced an increase in locomotor activity over time compared to the vehicle (Figure 2D,E, interaction, *p* < 0.0001). However, the modest stimulation induced by all doses of SR-15099 and SR-17018 was lower than that seen with the clinical analgesics, which can be visualized by comparing the maximally efficacious doses (Figure 2F). Since the timing of peak drug effects can reflect differential pharmacokinetic properties (fentanyl and oliceridine having a faster onset and clearance relative to morphine, SR-17018, and SR-15099 [10,16]), statistical analysis over time was not applied. No hyperactivity was observed in MOR knockout (MOR-KO) mice at the highest doses tested (Figure S1).

### 3.3. Sensitization following Chronic Opioid Exposure

**Figure 3:**
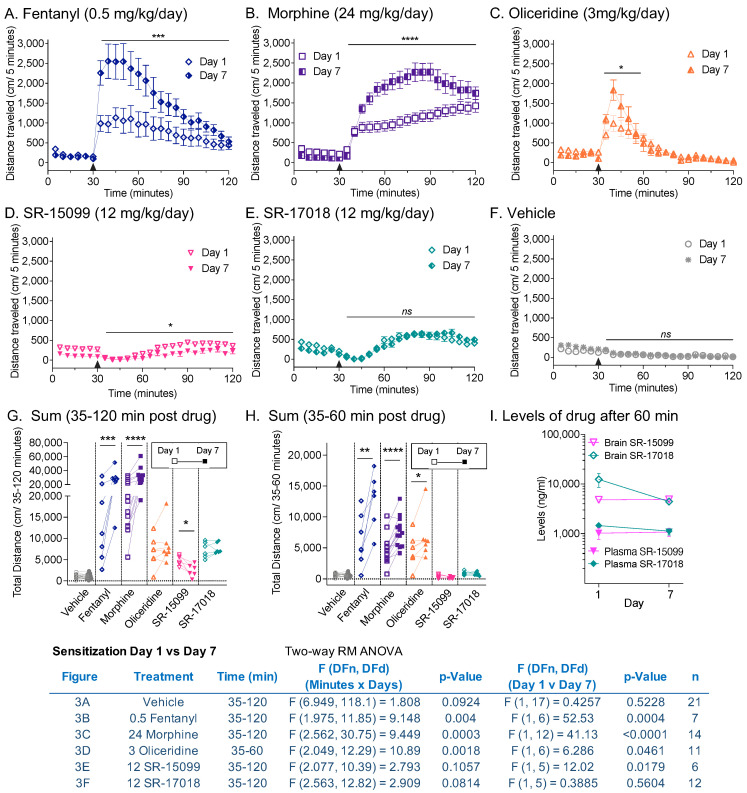
Repeated daily dosing of SR-17018 and SR-15099 did not lead to locomotor sensitization observed with fentanyl, morphine, and oliceridine. The mice from Figure 2 (at the corresponding drug and dose) were treated daily with (**A**) fentanyl (0.5 mg/kg, i.p.); (**B**) morphine (24 mg/kg, i.p.); (**C**) oliceridine (3 mg/kg, i.p.); (**D**) SR-15099 (12 mg/kg, i.p.); (**E**) SR-17018 (12 mg/kg, i.p.) or (**F**) vehicle. On day 7, mice were challenged i.p. with the same dose, and locomotor activity was assessed over 90 min following 30 min of habituation. The data are presented as mean ± SEM of the total distance (cm/5 min). (**C**) The sums of cumulative distance traveled (over 35–120 min) or (**D**) (over 35–60 min) after treatment, comparing Day 1 vs. Day 7, are presented. A statistical comparison over time using 2-way RM-ANOVA is indicated in (**A**–**F**) (see inserted table for statistics), and for (**G**,**H**) by paired Student’s *t*-test (* *p* < 0.05; ** *p* < 0.01 *** *p* < 0.001; **** *p* < 0.0001). (**I**) Brain and plasma levels of SR-17018 and SR-15099 on day 1 and day 7 of daily dosing (12 mg/kg, i.p.), taken 60 min after injection.

Daily drug administration was continued in the mice as shown in Figure 2 for 6 days at the indicated doses, and mice were again assessed for opioid-induced locomotor activity on the seventh day at the same dose (Figure 3). As anticipated, fentanyl (Figure 3A) and morphine (Figure 3B) produced enhanced responses after chronic dosing (day effect: fentanyl: *p* = 0.0004; morphine: *p* < 0.0001). Since oliceridine (Figure 3C) has a more transitory effect, due to its pharmacokinetic properties [18], the analyses were compared for the first 30 min after injection. During that period, sensitization was also observed following chronic daily dosing (day effect: *p* = 0.0461). Chronic treatment with SR-17018 (Figure 3D) and SR-15099 (Figure 3E) did not produce sensitization; moreover, daily administration of SR-15099 led to a modest, yet significant, decrease in locomotor activity on day 7 (day effect: *p* = 0.0179) over 90 min. Chronic vehicle treatment did not affect activity (Figure 3F). Individual mouse analyses for the sum of distance traveled is compared by one-way RM-ANOVA over 90 min (Figure 3G) and 30 min (Figure 3H) post-injection. Previously, we determined that high daily doses of SR-17018 (24 mg/kg) lead to precipitation at the site of injection [13]; therefore, we verified, as shown in Figure 3I, that the plasma and brain concentrations of SR-15099 and SR-17018 did not differ between the first and seventh days of testing (unpaired two-tailed *t* test), suggesting that the compounds had access to the brains of the mice, although no sensitization occurred.

### 3.4. SR-17018 Attenuates Morphine-Induced Locomotor Activity

**Figure 4:**
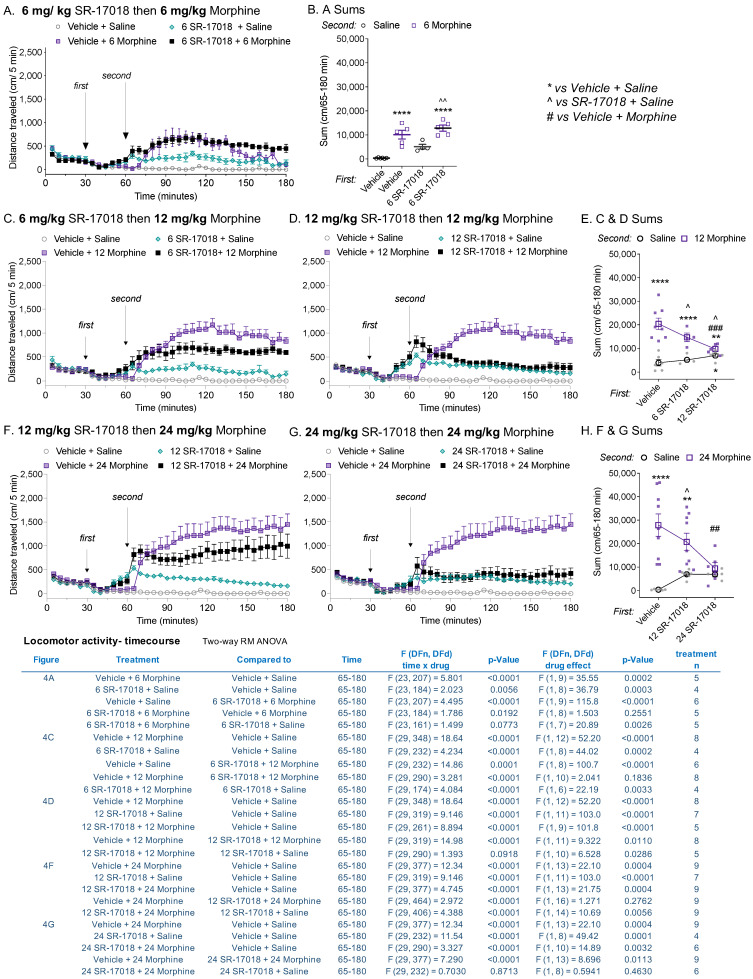
Pretreatment with SR-17018 dose-dependently attenuates morphine-induced locomotor activity. Mice were habituated for 30 min, then given 30 min of pretreatment (first) with either vehicle or SR-17018 prior to a challenge with saline or morphine (second); doses of each drug (IP) are indicated in the subheading for each figure. (**A**) Time course of SR-17018 (6 mg/kg), given before 6 mg/kg morphine, and (**B**) sum of total distance travelled, presented as mean ± SEM for individual mice. For the first injection, SR-17018 was given at (**C**) 6 mg/kg or (**D**) 12 mg/kg before 12 mg/kg morphine; (**E**) summarizes (**C**,**D**). For the first injection, SR17018 was given at (**F**) 12 mg/kg or (**G**) 24 mg/kg before 24 mg/kg morphine; (**H**) summarizes (**F**,**G**). Data are presented as raw locomotor activity along with the mean ± SEM of the total distance (cm) for (**A**,**C**–**F**) and the individual mouse sums for (**B**,**E**,**H**) with mean ± SEM. The inserted table presents a comparison of treatment effects over time, as determined by two-way RM-ANOVA; the sums were compared by ordinary one-way ANOVA (* *p* < 0.05; ** *p* < 0.01; **** *p* < 0.0001 vs. vehicle + saline; ^#^ *p* < 0.05, ^##^ *p* < 0.01, ^###^ *p* < 0.0001 vs. vehicle + morphine and ^ *p* < 0.05, ^^ *p* < 0.01 vs. SR-17018 + saline).

Since the peak effect of SR-17018 plateaus at a submaximal level relative to morphine, we asked what impact the combination of SR-17018 and morphine would have on the ambulatory behaviors of mice. Given that the SR-17018 brain distribution peaks between 30 and 120 min [10,13], mice were pretreated 30 min prior to morphine administration with SR-17018 or vehicle. SR-17018 (6 mg/kg, i.p) had no discernable impact on morphine (6 mg/kg, i.p.)-induced hyperactivity compared to morphine with vehicle pretreatment (Figure 4A, drug effect, *p* = 0.2550, Figure 4B for comparison of total distance). At higher doses of morphine, SR-17018 dose-dependently attenuated locomotor activity induced by morphine (Figure 4C–H; see inserted table for a summary of the pretreatment’s effect on statistical analyses of second treatments). Notably, the combination of SR-17018 and morphine produced a sustained lower level of hyperactivity, which remained consistently higher than the activity produced in the vehicle + saline-treated controls (Figure 4B,E,H: SR-17018 + morphine vs. vehicle + saline, ordinary one-way ANOVA: *p* < 0.0001).

To further investigate the nature of the inhibitory effect, we tested whether SR-17018 could modulate activity produced by a psychostimulant, *d*-amphetamine. A combination of opioids and psychostimulants typically potentiates hyperactivity in mice [19,20] due to the combination of converging mechanisms that induce dopamine release [20,21]. As anticipated, morphine pretreatment enhanced *d*-amphetamine-induced locomotor activity compared to vehicle pretreatment (Figure S2A, interaction of time and drug: *p* < 0.0001); pretreatment with SR-17018 also enhanced amphetamine-induced hyperactivity (Figure S2B, interaction of time and drug; *p* < 0.0001). These observations suggest that SR-17018 does not act by a MOR-independent means of sedating mice, and that it can still potentiate the effects of a stimulant such as morphine.

When administered 30 or 60 min after morphine, SR-17018 decreased morphine-induced hyperactivity (Figure S3A,B, two-way RM-ANOVA interaction of time and dose at 30 min (*p* = 0.0002); and at 60 min (*p* = 0.0032) post-treatment relative to morphine + vehicle; see table in Figure S3 for post hoc analysis). In all cases, SR-17018 decreased morphine-induced hyperlocomotion to a level approaching stimulation with SR-17018 alone (Figure S3A,B). On the other hand, naloxone fully antagonized morphine-induced locomotor activity to resemble saline + saline-induced activity levels (Figure S3C, two-way RM-ANOVA, interaction of drug and time: morphine + saline vs. morphine + naloxone: *p* < 0.0001; morphine + naloxone vs. saline + saline, *p* = 0.1106). This demonstrates an important difference between SR-17018 and naloxone, while naloxone serves as a competitive antagonist and blocks all of morphine’s effects, SR-17018 appears to act as a partial agonist in vivo, where its own activity is maintained while tempering the effects of morphine.

### 3.5. Co-Treatment of Partial Agonists Differentially Effects Morphine-Induced Hyperactivity and Antinociception

Oliceridine and SR-17018 act as partial agonists in the mouse striatum (Figure 1); however, only oliceridine acts as an orthosteric partial agonist, as it competes with DAMGO-stimulated ^35^S-GTPγS binding in the striatal membranes. Furthermore, we had previously shown this same pharmacological profile in the mouse brainstem (a region involved in descending pain perception) [15]. Therefore, we asked how the two agonists would interact with morphine in both the locomotor activity assay and the hot plate antinociception assay. Doses of each drug used were chosen based on the intent to produce a submaximal response in both the locomotor activity assay and the hot plate nociception assay to allow for the observation of an additive effect. Oliceridine has a rapid onset and relatively short metabolic life in mice [16,18]; therefore, we opted for a co-treatment approach.

**Figure 5:**
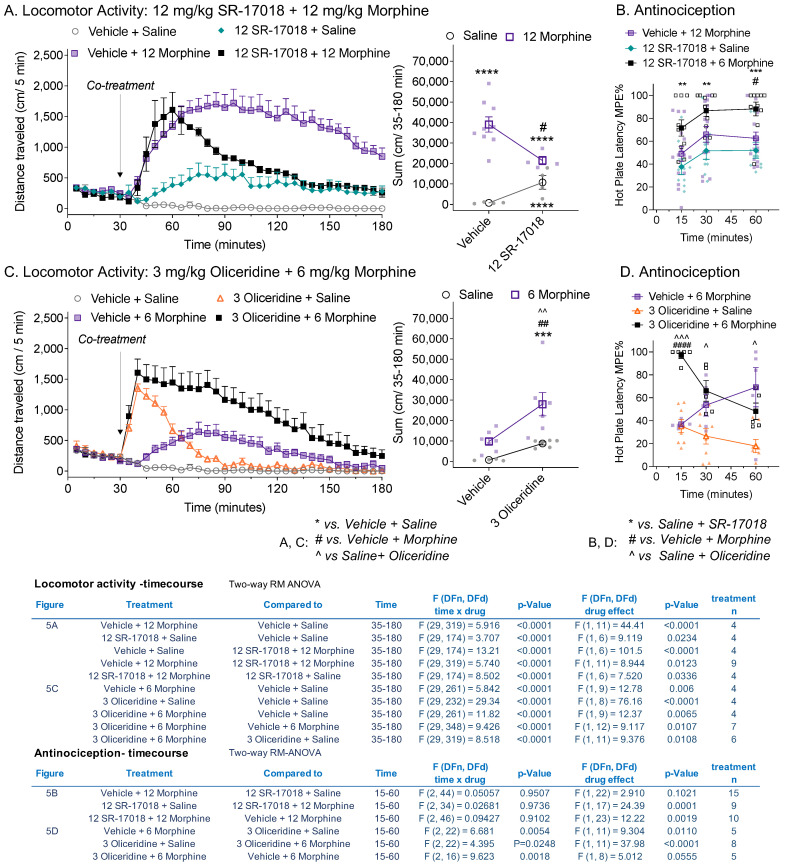
Co-treatment of SR-17018 with morphine decreases morphine-induced hyperactivity without attenuating antinociception. Comparison of in vivo drug interactions in the open field locomotor activity assay, showing total distance traveled (cm/5 min) (**left**) with total distance sums after co-treatment (cm/35–180 min) (**right**) and antinociception, assessing the latency to respond to the hot plate (52 °C) assay. (**A**) Distance traveled over time following co-administration with vehicle or SR-17018 (12 mg/kg, i.p.) and saline or morphine (12 mg/kg, i.p.), as indicated in the figure legend. (**B**) Hot plate response latency following the same drug and vehicle combinations, tested over 1 h at the indicated times (mean ± SEM). Vehicle + saline was not tested using the hot plate assay. (**C**) Distance traveled over time following co-administration of vehicle or oliceridine (3 mg/kg, i.p.) with saline or morphine (6 mg/kg, i.p.). (**D**) Hot plate response latency. For the distance traveled (**A**,**C**), individual mice and the mean ± SEM are shown, and a statistical comparison by two-way RM-ANOVA is presented in the inserted table. The sums of the distance traveled are compared by ordinary one-way ANOVA (*** *p* < 0.001; **** *p* < 0.0001 vs. vehicle + saline; ^#^ *p* < 0.05, ^##^ *p* < 0.01 vs. vehicle + morphine; ^^ *p* < 0.01 vs. SR-17018 + saline). For the antinociception studies (**B**,**D**) analysis over time is presented in the antinociception–time course table with two-way RM-ANOVA, while Šídák’s multiple comparisons post hoc analysis determined the significance: for (**C**): ** *p* < 0.01, *** *p* < 0.001 vs. SR-17018 + saline and ^#^ *p* < 0.05 vs. vehicle + morphine (**D**): ^ *p* < 0.05, ^^^ *p* < 0.001 vs. saline + oliceridine and ^####^ *p* < 0.0001 vs. vehicle + morphine.

As expected from the pre- and post- treatment studies (Figure 4 and Figure S3), co-treatment with SR-17018 suppressed morphine-induced hyperactivity (Figure 5A) (drug effect: vehicle + morphine vs. SR-17018 + morphine, *p* = 0.0123) to nearly the level of activity induced by SR-17018 alone. If the comparison was made 60 min following injection, allowing for SR-17018 drug delivery to the brain, the treatment groups did not differ (*p* = 0.2923), which was in agreement with the pretreatment studies (Figure 4). SR-17018 and morphine are equipotent in the hot plate test [10,13]. The combination of equi-efficacious doses of morphine and SR-17018 led to an increase in antinociceptive response compared to the effect of each drug alone in the hot plate assay (Figure 5B, drug effect: SR-17018 + morphine: vs. morphine + vehicle, *p* = 0.0019; vs. saline + SR-17018, *p* = 0.0001). This same effect was observed in female C57BL6 mice, in which SR-17018 attenuated morphine-induced locomotor activity while enhancing morphine-induced antinociception (Figure S5).

Oliceridine, when administered with morphine, produces more hyperactivity than either drug alone (Figure 5C, drug effect: oliceridine + morphine: vs. vehicle + morphine, *p* = 0.0107; vs. oliceridine + saline, *p* = 0.0108). Notably, we reduced the amount of morphine used in this assay, as the combination of 3 mg/kg oliceridine with 12 mg/kg morphine produced pronounced rigidity that interfered with the mouse’s ability to traverse the plastic surface of the activity box. Oliceridine at 3 mg/kg produced an equal antinociception effect of morphine at 6 mg/kg when dosed independently (Figure 5D); the combination of the two drugs significantly increased morphine-induced antinociception in the hot plate assay at the 15 min timepoint (Figure 5D). Over time, the antinociceptive effect of oliceridine appeared to wear off, in agreement with its short-lived bioactivity. However, the enhanced locomotor activity persisted for one hour post-injection, although oliceridine’s effects when administered with saline have waned by then (Figure 5C: drug effect from 90 to 180 min, comparing oliceridine + morphine: vs. vehicle + morphine, *p* = 0.0497; vs. oliceridine + saline, *p* = 0.0108).

## 4. Discussion

In this study, we explored partial agonism in vivo and in vitro using MOR-dependent behaviors and mouse striatal membranes, respectively. We provided dose–response curves of locomotor activity responses for direct comparison between biased MOR agonists and clinically used opioid analgesics, and also compared the intrinsic aspects of their efficacy in a relevant system. In mouse striatal membranes, all the tested compounds, including morphine and fentanyl, acted as partial agonists for inducing ^35^S-GTPγS binding (Figure 1), although their ability to promote hyperactivity varied (Figure 2). Oliceridine is a potent partial agonist in vitro that competes with DAMGO-stimulated ^35^S-GTPγS binding in the brain (current study; [15] for brainstem); yet, it also produces hyperactivity to the same extent as morphine and fentanyl (Figure 2 and Figure 3). Moreover, oliceridine also produces sensitization, as seen with morphine and fentanyl, while SR-17018 and SR-15099 do not (Figure 3). SR-17018 is also a partial agonist in the brain; however, it is noncompetitive with DAMGO-stimulated ^35^S-GTPγS coupling (current study (Figure 1); [15] for the brainstem). In the locomotor activity assay, SR-17018 produced a blunted hyperactivity response (Figure 2), and repeated dosing did not produce sensitization (Figure 3). The combination of SR-17018 and morphine decreased the hyperactivity produced by morphine while enhancing the observed antinociception (Figure 4 and Figure 5). When oliceridine and morphine were combined at submaximal doses, locomotor activity and antinociception were both enhanced (Figure 5).

Fentanyl, oliceridine, SR-17018, and SR-15099 have previously been described as biased agonists; while fentanyl has been shown to preferentially promote MOR-mediated βarrestin2 recruitment over G protein signaling, the other compounds are biased for G protein signaling [10,16]. In the mouse brain, all the tested compounds, including morphine and fentanyl, acted as partial agonists for inducing G protein signaling (^35^S-GTPγS binding) (Figure 1 and [10,15,22,23,24]). Recently, it was suggested that the improved therapeutic window (antinociception with less respiratory suppression) produced by oliceridine and SR-17018 is due to their low intrinsic efficacy at MOR as opposed to G protein signaling bias [11]. However, while fentanyl and morphine may appear to be highly efficacious agonists in frequently used receptor overexpression cellular systems, they are partial agonists with ~50% efficacy when tested in mouse brain tissue where there is low receptor reserve (Figure 1 for striatum, [10,15] for brainstem). Fentanyl and morphine also produce robust respiratory suppression in mice [25]. Buprenorphine, like oliceridine, is an agonist with low intrinsic efficacy in brain tissue and in cellular assays [23], and has been shown to produce morphine-like hyperactivity in ICR mice [26], (but not in Swiss Webster mice [27]); similarly, oliceridine also produces a robust hyperactivity response. Therefore, low intrinsic efficacy alone does not always lead to attenuated physiological responses and may not fully account for the attenuated hyperactivity produced by the SR series of opioid agonists.

In biochemical measures of intrinsic activity, partial agonists compete with full agonists for receptor occupancy; as a function of dose, the submaximal stimulation induced by the partial agonist overcomes the response produced by the full agonist as the partial agonist reaches full occupancy of the receptor population. As increasing concentrations of the partial agonist compete for occupancy, the signal produced by the full agonist will decreases and the partial agonist appears to have antagonistic properties. These observations can best be detected in assay systems that do not overly amplify the signaling output or have excessive receptor overexpression (i.e., spare receptors). The use of brain tissue to measure receptor signaling by ^35^S-GTPγS binding is a very relevant system by which to characterize agonist efficacy, as the receptor density of the mu opioid receptors is substantially lower than that of cellular overexpression systems [28]. In behavioral studies, this is rarely observed with opioids, as the combination of two partial agonists such as fentanyl and morphine will generally lead to an additive or synergistic effect [29]. One exception may be buprenorphine, which has antagonistic properties in the presence of other opioid agonists such as fentanyl and morphine, depending on the response and the system [30]. For example, buprenorphine produces a bell-shaped antinociceptive response in mice [31,32]; however, buprenorphine also has multiple receptor targets, and it becomes difficult to fully explain these effects by actions of the MOR alone [33].

A comparison of locomotor activity profiles revealed a dramatic difference between the SR-17018 and SR-15099 biased agonists and the other compounds. While they are were shown to produce hyperactivity, the profile captured an initial dip in activity, which was restored by 60 min post-injection. This ambulatory behavior remained remarkably steady over time, and while it was greater than that observed following vehicle treatment, it did not nearly reach the heights produced by the other agonists (Figure 2). In contrast, despite also being a G protein signaling-biased agonist, oliceridine produces a different profile, with the rapid-onset hyperactivity reaching peak effects similar to those of fentanyl (current study and [18]). At high doses, opioids lead to skeletal muscle rigidity in mice and humans, and this effect may contribute to cases of respiratory failure following overdose [34,35]. We recognize that high doses of opioids can lead to stiffness, which could impact our ability to measure the animal’s total distance traveled; a plateau effect can be observed for fentanyl in Figure 1, where a peak effect can be observed at 0.25 mg/kg, with higher doses producing no increase in distance. We considered this caveat when we repeatedly treated animals for sensitization using a sub-efficacious dose. Repeated dosing of fentanyl, morphine, and oliceridine produced sensitization (Figure 3), while SR-17018- and SR-15099-induced activity remained consistent with chronic administration (with a modest decrease in activity observed for SR-15099). Notably, prior analysis of SR-17018 and SR-15099 using the hot plate and tail flick nociceptive tests demonstrated that they are able to reach the same efficacy level as fentanyl and morphine, yet they do not produce respiratory suppression at doses exceeding their antinociceptive efficacy (48 mg/kg, i.p.) [10].

When combined with morphine, SR-17108 decreases the level of morphine-stimulated hyperactivity in a dose-dependent manner (Figure 4 and Figure 5). We hypothesize that this interaction may be due to SR-17018 promoting differential downstream signaling that does not result in the same behavioral output as that mediated by morphine [33]. SR-17018 was recently shown to be capable of binding within the orthosteric pocket [36] of MOR by cryo-electron microscopy studies. However, our prior study by Stahl et al., 2021 [15], reported radioligand competition binding and functional Schild analyses that supported a noncompetitive interaction between SR-17018 and MOR. In that study, we also showed that occupancy by SR-17018 stabilizes MOR-mediated GTPγS binding, which is wash-resistant (seems irreversible), but can be displaced by naloxone. Taken together, we favor a model wherein SR-17018 resides in close proximity to the receptor and has access to the orthosteric site where its occupancy favors MOR signaling to G protein (GTPγS binding). In the presence of SR-17018 (which appears to be resistant to washout), morphine has less access to the receptor; in this manner, we propose that the noncompetitive biased MOR agonist SR-17018 appears competitive with morphine in the locomotor activity assay. SR-17018 has been shown to lead to very little recruitment of βarrestin2 in cellular studies [10,36], and βarrestin2-KO mice demonstrates very little response to morphine compared to WT mice [8]; these correlations suggest that βarrestin2 may play some role in driving morphine-mediated hyperactivity in mice.

Locomotor hyperactivity and respiratory suppression are both affected by dopamine levels, which are elevated in response to typical opioid agonists [21,37]. It will be of interest to compare regional dopamine levels following the administration of these agonists in future studies and to delineate the rewarding and reinforcing properties of the novel SR G protein-biased MOR ligands. However, recent studies have shown that SR compounds are not devoid of conditioned place preference potential [38]. Their poor solubility limits their utility in self-administration studies, although Dr. Marc Caron (Duke University, Durham, NC, USA) attempted to use low doses in the mouse self-administration paradigm. Given the low dose, the lack of observed self-administration was not very telling regarding the abuse potential of the compound (unpublished observations). Still, chronic treatment with the novel MOR compounds may prevent some of the side effects of typical opioids. Upon chronic administration, SR-17018 produced no tolerance in the hot plate, formalin, or paclitaxel-induced neuropathy pain tests [13,14] and no sensitization to the locomotor stimulatory effects (Figure 3). Further, treatment of morphine-tolerant mice with SR-17018 suppressed withdrawal symptoms and restored morphine sensitivity with hot plate antinociception [13,38]; the ability to blunt morphine-induced psychomotor activity may also prove to be beneficial as an abuse deterrent. Further studies are needed to explore the potential of these novel compounds.

## Data Availability

Data are presented within the article and Supplementary materials. Raw values are plotted in the figures and are available upon request.

## Supplementary Materials

The following supporting information can be downloaded at: https://www.mdpi.com/article/10.3390/biom13060935/s1, Figure S1: A lack of opioid-induced hyperactivity in MOR-KO mice; Figure S2: SR-17018, like morphine, increases d-amphetamine-induced locomotor activity. Figure S3: SR-17018 reversal of morphine-induced locomotor activity; Figure S4: Co-treatment of morphine with SR-15099 attenuates morphine-induced hyperactivity; Figure S5: In female mice, co-treatment of morphine with SR-17018 decreases morphine-induced hyperactivity without attenuating antinociception.

## References

[1] Rethy C.R., Smith C.B., Villareal J.E. (1970). Effects of Narcotic Analgesics Upon the Locomotor Activity and Brain Cathecolamine Content of the Mouse. J. Pharmacol. Exp. Ther..

[2] Bailey A., Metaxas A., Al-Hasani R., Keyworth H.L., Forster D.M., Kitchen I. (2010). Mouse strain differences in locomotor, sensitisation and rewarding effect of heroin; Association with alterations in MOP-r activation and dopamine transporter binding. Eur. J. Neurosci..

[3] Varshneya N.B., Walentiny D.M., Moisa L.T., Walker T.D., Akinfiresoye L.R., Beardsley P.M. (2019). Opioid-like antinociceptive and locomotor effects of emerging fentanyl-related substances. Neuropharmacology.

[4] Hall F.S., Li X.F., Goeb M., Roff S., Hoggatt H., Sora I., Uhl G.R. (2003). Congenic C57BL/6 mu opiate receptor (MOR) knockout mice: Baseline and opiate effects. Genes Brain Behav..

[5] Sora I., Elmer G., Funada M., Pieper J., Li X.F., Hall F.S., Uhl G.R. (2001). Mu opiate receptor gene dose effects on different morphine actions: Evidence for differential in vivo mu receptor reserve. Neuropsychopharmacology.

[6] Bohn L.M., Lefkowitz R.J., Gainetdinov R.R., Peppel K., Caron M.G., Lin F.-T. (1999). Enhanced Morphine Analgesia in Mice Lacking Beta-Arrestin 2. Science.

[7] Bohn L.M., Gainetdinov R.R., Lin F.T., Lefkowitz R.J., Caron M.G. (2000). Mu-opioid receptor desensitization by beta-arrestin-2 determines morphine tolerance but not dependence. Nature.

[8] Bohn L.M., Gainetdinov R.R., Sotnikova T.D., Medvedev I.O., Lefkowitz R.J., Dykstra L.A., Caron M.G. (2003). Enhanced rewarding properties of morphine, but not cocaine, in beta(arrestin)-2 knock-out mice. J. Neurosci..

[9] Mittal N., Tan M., Egbuta O., Desai N., Crawford C., Xie C.W., Evans C., Walwyn W. (2012). Evidence that behavioral phenotypes of morphine in beta-arr2−/− mice are due to the unmasking of JNK signaling. Neuropsychopharmacology.

[10] Schmid C.L., Kennedy N.M., Ross N.C., Lovell K.M., Yue Z., Morgenweck J., Cameron M.D., Bannister T.D., Bohn L.M. (2017). Bias Factor and Therapeutic Window Correlate to Predict Safer Opioid Analgesics. Cell.

[11] Gillis A., Gondin A.B., Kliewer A., Sanchez J., Lim H.D., Alamein C., Manandhar P., Santiago M., Fritzwanker S., Schmiedel F. (2020). Low intrinsic efficacy for G protein activation can explain the improved side effect profiles of new opioid agonists. Sci. Signal..

[12] Cornelissen J.C., Blough B.E., Bohn L.M., Negus S.S., Banks M.L. (2021). Some effects of putative G-protein biased mu-opioid receptor agonists in male rhesus monkeys. Behav. Pharmacol..

[13] Grim T.W., Schmid C.L., Stahl E.L., Pantouli F., Ho J.H., Acevedo-Canabal A., Kennedy N.M., Cameron M.D., Bannister T.D., Bohn L.M. (2019). A G protein signaling-biased agonist at the mu-opioid receptor reverses morphine tolerance while preventing morphine withdrawal. Neuropsychopharmacology.

[14] Pantouli F., Grim T.W., Schmid C.L., Acevedo-Canabal A., Kennedy N.M., Cameron M.D., Bannister T.D., Bohn L.M. (2021). Comparison of morphine, oxycodone and the biased MOR agonist SR-17018 for tolerance and efficacy in mouse models of pain. Neuropharmacology.

[15] Stahl E.L., Schmid C.L., Acevedo-Canabal A., Read C., Grim T.W., Kennedy N.M., Bannister T.D., Bohn L.M. (2021). G protein signaling-biased mu opioid receptor agonists that produce sustained G protein activation are noncompetitive agonists. Proc. Natl. Acad. Sci. USA.

[16] DeWire S.M., Yamashita D.S., Rominger D.H., Liu G., Cowan C.L., Graczyk T.M., Chen X.T., Pitis P.M., Gotchev D., Yuan C. (2013). A G protein-biased ligand at the mu-opioid receptor is potently analgesic with reduced gastrointestinal and respiratory dysfunction compared with morphine. J. Pharmacol. Exp. Ther..

[17] Zhou L., Stahl E.L., Lovell K.M., Frankowski K.J., Prisinzano T.E., Aube J., Bohn L.M. (2015). Characterization of kappa opioid receptor mediated, dynorphin-stimulated [35S]GTPgammaS binding in mouse striatum for the evaluation of selective KOR ligands in an endogenous setting. Neuropharmacology.

[18] Mori T., Takemura Y., Arima T., Iwase Y., Narita M., Miyano K., Hamada Y., Suda Y., Matsuzawa A., Sugita K. (2021). Further investigation of the rapid-onset and short-duration action of the G protein-biased mu-ligand oliceridine. Biochem. Biophys. Res. Commun..

[19] Mori T., Ito S., Narita M., Suzuki T., Sawaguchi T. (2004). Combined effects of psychostimulants and morphine on locomotor activity in mice. J. Pharmacol. Sci..

[20] Trujillo K.A., Smith M.L., Guaderrama M.M. (2011). Powerful behavioral interactions between methamphetamine and morphine. Pharmacol. Biochem. Behav..

[21] Di Chiara G., Imperato A. (1988). Drugs abused by humans preferentially increase synaptic dopamine concentrations in the mesolimbic system of freely moving rats. Proc. Natl. Acad. Sci. USA.

[22] Selley D.E., Sim L.J., Xiao R., Liu Q., Childers S.R. (1997). μ-Opioid Receptor-Stimulated Guanosine-5′-O-(γ-thio)-triphosphate Binding in Rat Thalamus and Cultured Cell Lines: Signal Transduction Mechanisms Underlying Agonist Efficacy. Mol. Pharmacol..

[23] Selley D.E., Liu Q., Childers S.R. (1998). Signal transduction correlates of mu opioid agonist intrinsic efficacy: Receptor-stimulated [35S]GTP gamma S binding in mMOR-CHO cells and rat thalamus. J. Pharmacol. Exp. Ther..

[24] Lester P.A., Traynor J.R. (2006). Comparison of the in vitro efficacy of mu, delta, kappa and ORL1 receptor agonists and non-selective opioid agonists in dog brain membranes. Brain. Res..

[25] Varshneya N.B., Hassanien S.H., Holt M.C., Stevens D.L., Layle N.K., Bassman J.R., Iula D.M., Beardsley P.M. (2022). Respiratory depressant effects of fentanyl analogs are opioid receptor-mediated. Biochem. Pharmacol..

[26] Santos E.J., Banks M.L., Negus S.S. (2022). Role of Efficacy as a Determinant of Locomotor Activation by Mu Opioid Receptor Ligands in Female and Male Mice. J. Pharmacol. Exp. Ther..

[27] Varshneya N.B., Walentiny D.M., Stevens D.L., Walker T.D., Akinfiresoye L.R., Beardsley P.M. (2023). Structurally diverse fentanyl analogs yield differential locomotor activities in mice. Pharmacol. Biochem. Behav..

[28] Sim-Selley L.J., Scoggins K.L., Cassidy M.P., Smith L.A., Dewey W.L., Smith F.L., Selley D.E. (2007). Region-dependent attenuation of mu opioid receptor-mediated G-protein activation in mouse CNS as a function of morphine tolerance. Br. J. Pharmacol..

[29] Romero A., Miranda H.F., Puig M.M. (2010). Analysis of the opioid-opioid combinations according to the nociceptive stimulus in mice. Pharmacol. Res..

[30] Varshneya N.B., Thakrar A.P., Hobelmann J.G., Dunn K.E., Huhn A.S. (2022). Evidence of Buprenorphine-precipitated Withdrawal in Persons Who Use Fentanyl. J. Addict. Med..

[31] Lutfy K., Eitan S., Bryant C.D., Yang Y.C., Saliminejad N., Walwyn W., Kieffer B.L., Takeshima H., Carroll F.I., Maidment N.T. (2003). Buprenorphine-induced antinociception is mediated by mu-opioid receptors and compromised by concomitant activation of opioid receptor-like receptors. J. Neurosci..

[32] Khroyan T.V., Wu J., Polgar W.E., Cami-Kobeci G., Fotaki N., Husbands S.M., Toll L. (2014). BU08073 a buprenorphine analogue with partial agonist activity at mu-receptors in vitro but long-lasting opioid antagonist activity in vivo in mice. Br. J. Pharmacol..

[33] Acevedo-Canabal A., Pantouli F., Ravichandran A., Rullo L., Bohn L.M., Kenakin T. (2022). 3.21—Pharmacological Diversity in Opioid Analgesics: Lessons From Clinically Useful Drugs. Comprehensive Pharmacology.

[34] Torralva R., Janowsky A. (2019). Noradrenergic Mechanisms in Fentanyl-Mediated Rapid Death Explain Failure of Naloxone in the Opioid Crisis. J. Pharmacol. Exp. Ther..

[35] Hill R., Santhakumar R., Dewey W., Kelly E., Henderson G. (2020). Fentanyl depression of respiration: Comparison with heroin and morphine. Br. J. Pharmacol..

[36] Zhuang Y., Wang Y., He B., He X., Zhou X.E., Guo S., Rao Q., Yang J., Liu J., Zhou Q. (2022). Molecular recognition of morphine and fentanyl by the human mu-opioid receptor. Cell.

[37] Lalley P.M. (2008). Opioidergic and dopaminergic modulation of respiration. Respir. Physiol. Neurobiol..

[38] Kudla L., Bugno R., Podlewska S., Szumiec L., Wiktorowska L., Bojarski A.J., Przewlocki R. (2022). Comparison of an Addictive Potential of μ-Opioid Receptor Agonists with G Protein Bias: Behavioral and Molecular Modeling Studies. Pharmaceutics.

