# Supplementary material for: Hyperactivity in Mice Induced by Opioid Agonists with Partial Intrinsic Efficacy and Biased Agonism Administered Alone and in Combination with Morphine
Source: Biomolecules. 2023 Jun 2;13(6):935. doi: 10.3390/biom13060935 (PMC10295947; doi:10.3390/biom13060935)
Supplement: Supplementary file 1 [file biomolecules-13-00935-s001.zip › biomolecules-2356947-supplementary.pdf]

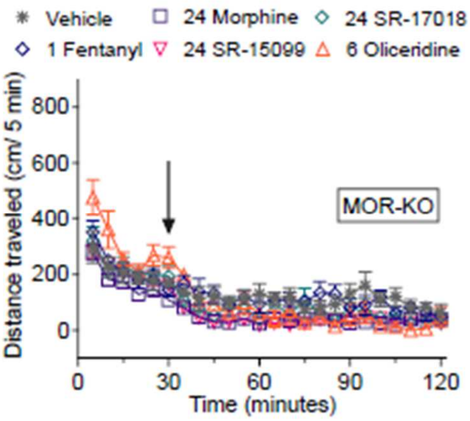

MOR-KO drug vs vehicle | **Two-way RM ANOVA**

Figure	Drug vs vehicle	Time (min)	F (DFn, DFd) Time x Drug	p-Value	F (DFn, DFd) Drug v Vehicle	p-Value	Difference	SE	Drug treatment n
SF1A	1 Fentanyl	35-120	F (17, 204) = 1.284	P=0.2055	F (1, 12) = 0.02158	P=0.8857	4.46	30.36	7
	24 Morphine	35-120	F (17, 272) = 1.151	P=0.3055	F (1, 16) = 8.927	P=0.0087	-66.85	22.37	7
	6 Oliceridine	35-120	F (17, 221) = 2.231	P=0.0044	F (1, 13) = 2.472	P=0.1399	-46.65	29.67	4
	24 SR-15099	35-60	F (17, 340) = 1.281	P=0.2016	F (1, 20) = 13.60	P=0.0015	-68.43	18.56	11
	24 SR-17018	35-120	F (17, 221) = 0.8418	P=0.6436	F (1, 13) = 1.856	P=0.1962	-40.36	29.63	4

vehicle: n=11

Figure S1. A lack of opioid-induced hyperactivity in MOR-KO mice. Male MOR-KO (n = 4–11) were IP treated with vehicle or the highest dose of each opioid tested after a 30-minute habituation period. Distance traveled (cm/5-minute bins) for 90 minutes was recorded and no locomotor stimulation was observed in the MOR-KO. Data are presented as mean ◎ SEM.

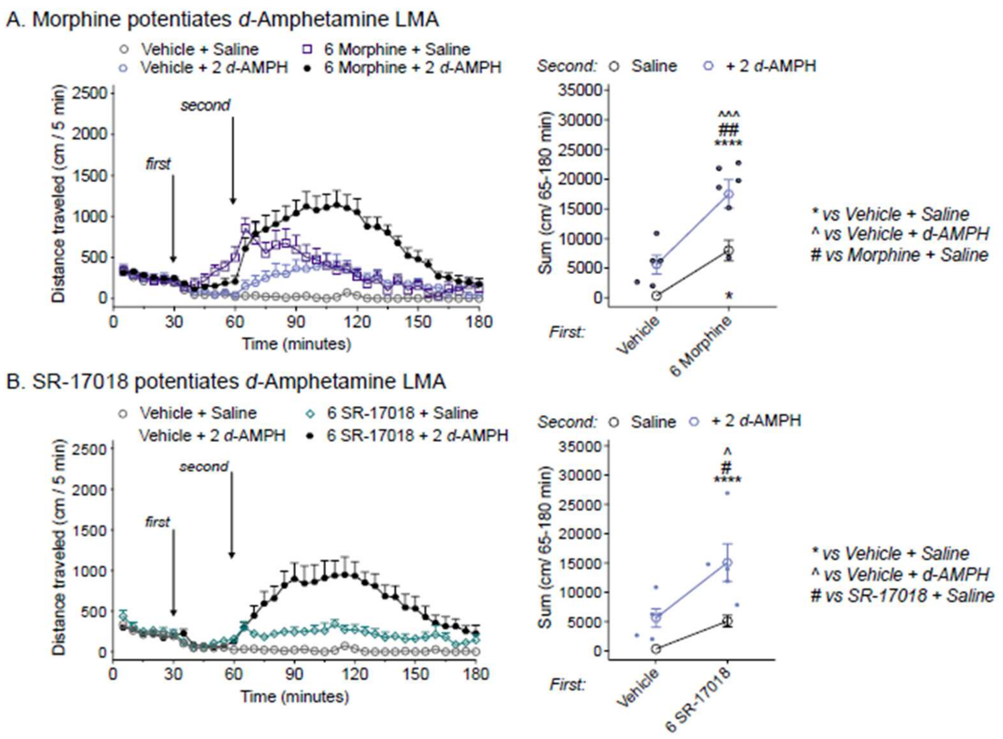

Locomotor Activity

TIMECOURSE Two-way RM ANOVA

Figure	Treatment	Compared to	Time	F (DFn, DFd) time x drug	p-Value	F (DFn, DFd) drug effect	p-Value	treatment n
SF2A	Vehicle + d-Amphetamine	Vehicle + Saline	65-180	F (29, 261) = 7.363	<0.0001	F (1, 9) = 12.34	0.0066	5
	6 Morphine + Saline	Vehicle + Saline	65-180	F (29, 232) = 10.67	<0.0001	F (1, 8) = 30.45	0.0006	4
	6 Morphine + d-Amphetamine	Vehicle + Saline	65-180	F (29, 290) = 20.05	<0.0001	F (1, 10) = 50.83	<0.0001	5
	Vehicle + d-Amphetamine	6 Morphine + d-Amphetamine	65-180	F (29, 261) = 6.634	<0.0001	F (1, 9) = 15.35	0.0035	5
	6 Morphine + Saline	6 Morphine + d-Amphetamine	65-180	F (29, 232) = 7.194	<0.0001	F (1, 8) = 6.193	0.0376	5
SF2B	Vehicle + d-Amphetamine	Vehicle + Saline	65-180	F (29, 261) = 7.363	<0.0001	F (1, 9) = 12.34	0.0066	8
	6 SR-17018 + Saline	Vehicle + Saline	65-180	F (29, 232) = 4.234	<0.0001	F (1, 8) = 44.02	0.0002	4
	6 SR-17018 + d-Amphetamine	Vehicle + Saline	65-180	F (29, 261) = 14.73	<0.0001	F (1, 9) = 27.44	0.0005	7
	Vehicle + d-Amphetamine	6 SR-17018 + d-Amphetamine	65-180	F (29, 232) = 3.882	<0.0001	F (1, 8) = 7.116	0.0285	4
	6 SR-17018 + Saline	6 SR-17018 + d-Amphetamine	65-180	F (29, 203) = 5.484	<0.0001	F (1, 7) = 7.291	0.0306	5

SUMS Ordinary one-way ANOVA, Šídák's multple comparison post-hoc

Figure	Treatment	Compared to	Time	p-Value	treatment n	
SF2A	Vehicle + d-Amphetamine	Vehicle + Saline	65-180	0.1825	5	ns
	6 Morphine + Saline	Vehicle + Saline	65-180	0.0375	4	*
	6 Morphine + d-Amphetamine	Vehicle + Saline	65-180	<0.0001	5	****
	Vehicle + d-Amphetamine	6 Morphine + d-Amphetamine	65-180	0.0005	5	^^^
	6 Morphine + Saline	6 Morphine + d-Amphetamine	65-180	0.0075	5	##
SF2B	Vehicle + d-Amphetamine	Vehicle + Saline	65-180	0.2198	8	ns
	6 SR-17018 + Saline	Vehicle + Saline	65-180	0.3739	4	ns
	6 SR-17018 + d-Amphetamine	Vehicle + Saline	65-180	<0.0001	7	****
	Vehicle + d-Amphetamine	6 SR-17018 + d-Amphetamine	65-180	0.0102	4	^
	6 SR-17018 + Saline	6 SR-17018 + d-Amphetamine	65-180	0.0107	5	#

Figure S2. SR-17018, like, morphine, increases d-amphetamine-induced locomotor activity. **(A,B)**. Pretreatment with the vehicle, morphine (6 mg/kg, i.p.), or SR-17018 (6 mg/kg, i.p.) for 30 minutes followed by challenge with saline or *d*-amphetamine (2 mg/kg, i.p.) in male C57BL6 mice (n=4-6). Effects over time *(left)* and sum of the distance traveled (cm) from 65-180 minutes for individual mice *(right)* are shown in (**A**,**B**). Comparison of sums are indicated in the figure. Data are presented as mean ◎ SEM of the total distance (cm).

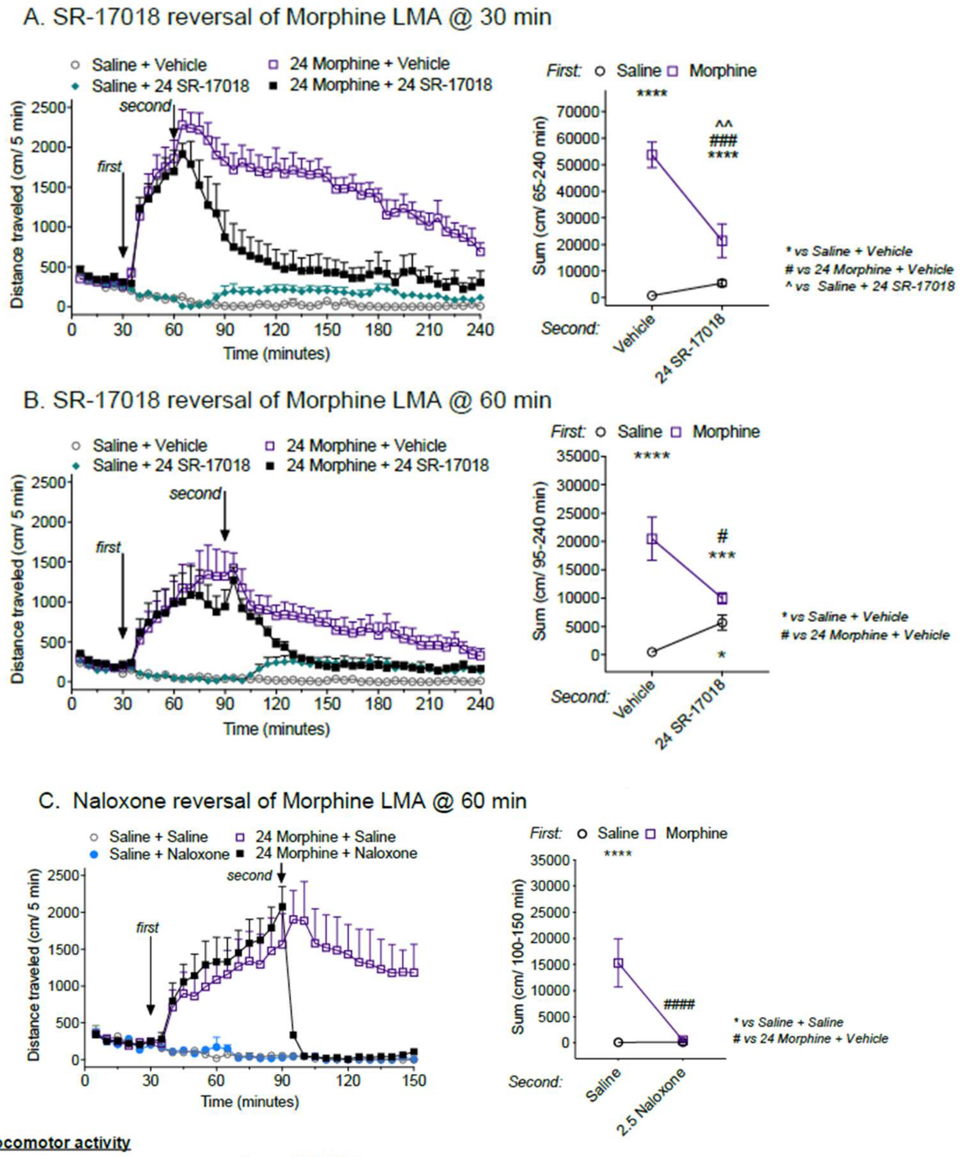

Locomotor activity

Timecourse: Two-way RM ANOVA

Figure	Treatment	Compared to	Time	F (DFn, DFd) time x drug	p-Value	F (DFn, DFd) drug effect	p-Value	treatment n
SF3A	24 Morphine + Vehicle	Saline + Vehicle	65-240	F (35, 350) = 7.374	<0.0001	F (1, 10) = 83.36	<0.0001	7
	Saline + 24 SR-17018	Saline + Vehicle	65-240	F (35, 350) = 2.886	<0.0001	F (1, 10) = 11.04	0.0077	7
	Saline + Vehicle	24 Morphine + 24 SR-17018	65-240	F (35, 280) = 12.94	<0.0001	F (1, 8) = 10.67	0.0114	5
	24 Morphine + Vehicle	24 Morphine + 24 SR-17018	65-240	F (35, 350) = 2.192	0.0002	F (1, 10) = 17.14	0.002	7
	24 Morphine + 24 SR-17018	Saline + 24 SR-17018	65-240	F (35, 350) = 20.51	<0.0001	F (1, 10) = 8.687	0.0146	5
SF3B	24 Morphine + Vehicle	Saline + Vehicle	95-240	F (29, 290) = 4.476	<0.0001	F (1, 10) = 13.19	0.0046	7
	Saline + 24 SR-17018	Saline + Vehicle	95-240	F (29, 174) = 3.693	<0.0001	F (1, 6) = 15.24	0.0079	7
	Saline + Vehicle	24 Morphine + 24 SR-17018	95-240	F (29, 290) = 13.26	<0.0001	F (1, 10) = 48.75	<0.0001	5
	24 Morphine + Vehicle	24 Morphine + 24 SR-17018	95-240	F (29, 406) = 1.924	0.0032	F (1, 14) = 7.235	0.0176	7
	24 Morphine + 24 SR-17018	Saline + 24 SR-17018	95-240	F (29, 290) = 17.39	<0.0001	F (1, 10) = 7.040	0.0242	5
SF3C	Saline + 2.5 Naloxone	Saline + Saline	100-150	F (10, 60) = 0.6125	0.7971	F (1, 6) = 0.3596	0.5706	4
	24 morphine + Saline	Saline + Saline	100-150	F (10, 80) = 2.870	0.0042	F (1, 8) = 6.920	0.0302	6
	Saline + Saline	24 morphine + 2.5 naloxone	100-150	F (10, 80) = 1.639	0.1106	F (1, 8) = 6.970	0.0297	4
	24 morphine + 2.5 naloxone	24 morphine + Saline	100-150	F (10, 100) = 5.270	<0.0001	F (1, 10) = 10.28	0.0094	6
	24 morphine + 2.5 naloxone	Saline + 2.5 Naloxone	100-150	F (10, 80) = 1.101	0.3717	F (1, 8) = 5.600	0.0455	6

SUMS Ordinary one-way ANOVA, Šidák's multple comparison post-hoc

Figure	Treatment	Compared to	Time	p-Value	treatment n	
SF3A	24 Morphine + Vehicle	Saline + Vehicle	65-240	0.9313	7	ns
	Saline + 24 SR-17018	Saline + Vehicle	65-240	<0.0001	7	****
	Saline + Vehicle	24 Morphine + 24 SR-17018	65-240	0.014	5	*
	24 Morphine + Vehicle	24 Morphine + 24 SR-17018	65-240	0.0491	7	*
	24 Morphine + 24 SR-17018	Saline + 24 SR-17018	65-240	<0.0001	5	****
SF3B	24 Morphine + Vehicle	Saline + Vehicle	95-240	0.0004	7	***
	Saline + 24 SR-17018	Saline + Vehicle	95-240	0.8083	7	ns
	Saline + Vehicle	24 Morphine + 24 SR-17018	95-240	0.1414	5	ns
	24 Morphine + Vehicle	24 Morphine + 24 SR-17018	95-240	0.0235	7	*
	24 Morphine + 24 SR-17018	Saline + 24 SR-17018	95-240	0.8354	5	ns
SF3C	Saline + 2.5 Naloxone	Saline + Saline	100-150	>0.9999	4	ns
	24 morphine + Saline	Saline + Saline	100-150	0.0093	6	**
	Saline + Saline	24 morphine + 2.5 naloxone	100-150	>0.9999	4	ns
	24 morphine + 2.5 naloxone	24 morphine + Saline	100-150	0.0046	6	**
	24 morphine + 2.5 naloxone	Saline + 2.5 Naloxone	100-150	>0.9999	6	ns

Figure S3. SR-17018 reversal of morphine-induced locomotor activity. The basal activity was recorded for 30 minutes followed by 30- (**A**) or 60-minute (**B**) treatment with saline or morphine (24 mg/kg, i.p.) before vehicle

or SR-17018 (24 mg/kg, i.p.) challenge in male C57BL6 mice (n = 4–8). Effects over time *(left)* and sum or the distance traveled (cm) from 65-240 minutes for individual mice *(right)* are shown in (**A–B**). **C**. Naloxone (2.5 mg/kg, i.p.) reversal after 60 minutes of morphine locomotor activity is shown for reference (n = 4-6). Comparison of sums are indicated in the figure. Sums figures have different Y-axis scales based on the maximal stimulation per treatment combination and we are showing individual sums per mouse. **D**. Table showing least square mean differences Tukey HSD analysis for pretreatment x second treatment interactions after saline or morphine + vehicle, SR-17018, or naloxone challenge. Data are presented as mean ± SEM of the total distance (cm).

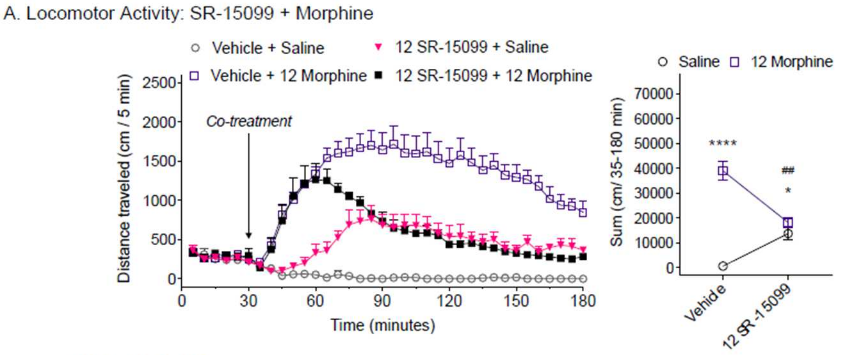

Locomotor activity

Timecourse: Two-way RM ANOVA

Figure	Treatment	Compared to	Time	F (DFn, DFd) (Minutes x Days)	p-Value	F (DFn, DFd) (Day 1 v Day 7)	p-Value	treatment n
SF5	Vehicle + 12 Morphine	Vehicle + Saline	35-180	F (29, 319) = 5.916	<0.0001	F (1, 11) = 44.41	<0.0001	4
	12 SR-15099 + Saline	Vehicle + Saline	35-180	F (29, 174) = 8.913	<0.0001	F (1, 6) = 25.76	0.0023	4
	12 SR-15099 + 12 Morphine	Vehicle + Saline	35-180	F (29, 174) = 15.95	<0.0001	F (1, 6) = 74.92	0.0001	4
	Vehicle + 12 Morphine	12 SR-15099 + 12 Morphine	35-180	F (29, 319) = 4.950	<0.0001	F (1, 11) = 12.43	0.0047	9
	12 SR-15099 + 12 Morphine	12 SR-15099 + Saline	35-180	F (29, 174) = 11.98	<0.0001	F (1, 6) = 1.830	0.2249	4

SUMS Ordinary one-way ANOVA, Šidák's multple comparison post-hoc

Figure	Treatment	Compared to	Time	p-Value	treatment n	
SF5	Vehicle + 12 Morphine	Vehicle + Saline	35-180	<0.0001	4	****
	12 SR-15099 + Saline	Vehicle + Saline	35-180	0.1695	4	ns
	12 SR-15099 + 12 Morphine	Vehicle + Saline	35-180	0.0366	4	*
	Vehicle + 12 Morphine	12 SR-15099 + 12 Morphine	35-180	0.0028	9	**
	12 SR-15099 + 12 Morphine	12 SR-15099 + Saline	35-180	0.9513	4	ns

Figure S4. Co-treatment of morphine with SR-15099 attenuates morphine-induced hyperactivity. *In vivo* drug interactions in the open field locomotor activity assay showing total distance traveled (cm/ 5-minutes) *(left)* with total distance sums after co-treatment (cm/ 35-180 minutes) (*right*) in male C57BL/6 (n = 4–7). Locomotor activity after cotreatment with vehicle or SR-15099 (12 mg/kg, i.p.) and saline or morphine (12 mg/kg, i.p.). SR-15099 and morphine drug combination attenuate morphine's hyperactivity to the level of SR-15099. Comparison of sums are shown in the figure. Data are presented as mean ± SEM.

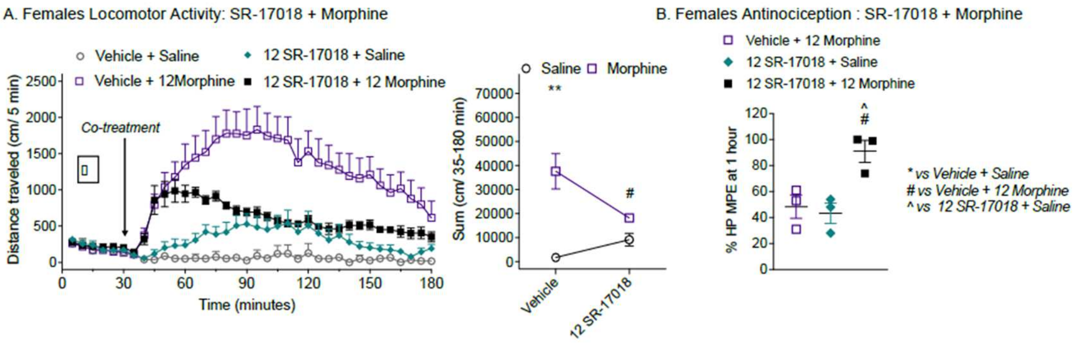

Locomotor Activity

TIMECOURSE **Two-way RM ANOVA**

Figure	Treatment	Compared to	Time	F (DFn, DFd) time x drug	p-Value	F (DFn, DFd) drug effect	p-Value	treatment n
SF4a	Vehicle + 12 Morphine	Vehicle + Saline	35-180	F (29, 203) = 6.199	P<0.0001	F (1, 7) = 11.12	P=0.0125	6
	12 SR-17018 + Saline	Vehicle + Saline	35-180	F (29, 145) = 2.521	P=0.0002	F (1, 5) = 5.285	P=0.0699	4
	12 SR-17018 + 12 Morphine	Vehicle + Saline	35-180	F (29, 290) = 7.029	P<0.0001	F (1, 10) = 6.869	P=0.0256	6
	Vehicle + 12 Morphine	12 SR-17018 + 12 Morphine	35-180	F (29, 290) = 7.029	P<0.0001	F (1, 10) = 6.869	P=0.0256	6
	12 SR-17018 + 12 Morphine	12 SR-17018 + Saline	35-180	F (29, 232) = 4.238	P<0.0001	F (1, 8) = 13.14	P=0.0067	6

Sums **Ordinary one-way ANOVA, Šidák's multple comparison post-hoc**

Figure	Treatment	Compared to	Time	p-Value	treatment n	
SF4a	Vehicle + 12 Morphine	Vehicle + Saline	35-180	0.0014	6	**
	12 SR-17018 + Saline	Vehicle + Saline	35-180	0.9099	4	ns
	12 SR-17018 + 12 Morphine	Vehicle + Saline	35-180	0.216	6	ns
	Vehicle + 12 Morphine	12 SR-17018 + 12 Morphine	35-180	0.0342	6	*
	12 SR-17018 + 12 Morphine	12 SR-17018 + Saline	35-180	0.699	6	ns

vehicle+saline n=3

Hot Plate Latency

Ordinary one-way ANOVA, Šidák's multple comparison post-hoc

	Treatment	Compared to	Time	p-Value	treatment n	
SF4b	Vehicle + 12 Morphine	12 SR-17018 + Saline	1h	0.90960	3	
	12 SR-17018 + 12 Morphine	Vehicle + 12 Morphine	1h	0.0274	3	#
	12 SR-17018 + 12 Morphine	12 SR-17018 + Saline	1h	0.017	3	^

Figure S5. In female mice, co-treatment of morphine with SR-17018 decreases morphine-induced hyperactivity without attenuating antinociception. Results recapitulate in female C57BL6 mice testing for SR-17018 + morphine locomotor (n = 3–6) and antinociception (n = 3) Doses are indicated as mg/kg, i.p. in the figure legends. Data are presented as mean ◎SEM. .
